# Viral infection at the endothelium

**DOI:** 10.18632/oncotarget.5246

**Published:** 2015-08-22

**Authors:** Felicia Goodrum, Farah Bughio

**Affiliations:** Department of Cellular and Molecular Medicine, Department of Immunology, and BIO5 Institute, The University of Arizona, Tucson, Arizona, USA

**Keywords:** cytomegalovirus, herpesvirus, endothelial, vascular disease

Vascular endothelial cells (ECs) line the inner surface of blood vessels, providing a critical barrier between the vasculature and organ systems. ECs represent an important target for infection of most human viruses, including beta- and gamma-herpesviruses. Infection of the endothelium has profound implications for both the virus and the host. For the virus, infection of ECs can provide a gateway for dissemination to organs and a reservoir for long-term persistence. For the host, virus replication and the ensuing immune response at the endothelium increases tissue permeability and inflammation, contributing to vascular and pulmonary diseases and to the severity of viral disease. Despite the clear importance of the vascular endothelium in viral infection, we know little about infection in ECs.

Human cytomegaloviruses (CMV), the ubiquitous and prototypical β-herpesvirus, is remarkable in its ability to infect a wide variety of cell types resulting in a complex persistence in the host. CMV productively replicates in some cell types, such as fibroblasts, and is latent in hematopoietic progenitor and myeloid-lineage cells. The latent infection is defined by the maintenance of viral genomes, but replication is reversibly abated. Still other cell types are sites of long-term virus shedding as they support a chronic infection (endothelial and epithelial) where low levels of virus are produced over very long time periods. Cell intrinsic factors influence the ultimate outcome of infection: replicative, chronic, and latent. Specialized cell types provide unique environments for and barriers to infection that CMV must negotiate to ensure persistence.

CMV persistence is marked by sporadic and subclinical reactivation events in healthy individuals. In the immunocompromised, reactivation of CMV from latency causes life-threatening disease characterized by pneumonitis, retinitis, gastroenteritis, and hepatitis. CMV, the “troll of transplantation”, is particularly problematic in solid organ and stem cell transplantation. In addition to the risk of CMV disease, CMV is an important risk factor in graft failure and the long-term outcome for transplant recipients. While once considered benign, important long-term costs of subclinical viral persistence are emerging. CMV sero-positivity in healthy individuals carries an increased risk of vascular disease, including atherosclerosis, frailty, and cardiovascular mortality. CMV infection of ECs promotes proinflammatory signaling contributing to angiogenesis and vascular disease [1–4]. The collective data indicates that CMV infection-both its persistence in healthy individuals and its disease in transplant patients-presents a significant risk with profound implications for vascular health.

Clinical isolates of CMV replicate with differing efficiencies in ECs—suggesting the existence of viral determinants important for tropism in ECs beyond virus entry. To identify these viral genes, we generated and screened a library of recombinant viruses containing disruptions in the UL*b*’ region of the HCMV genome. We focused on the UL*b*’ region because it is retained in clinical strains but uniformly lost during serial virus passage in cultured fibroblasts, suggesting that these genes are required for replication in other cell types or for persistence in the host. We identified the *UL133-UL138* (*UL133/8*) polycistronic locus as important for replication in ECs [5, 6]. Infection of ECs with a recombinant virus lacking the locus results in a disorganization of secretory membranes, a failure to incorporate virus into multivesicular bodies (MVBs), and defects in virion maturation [5]. MVBs are unique organelles at the center of endo-/exocytic pathways that play a critical role in sorting cellular components for degradation in lysosomes or exocytosis. Further, MVBs are also platforms for modulating signaling in the cell. These phenotypes indicate a critical role of UL133/8 proteins in co-opting cellular trafficking pathways for replication, viral maturation and egress in ECs.

**Figure 1:**
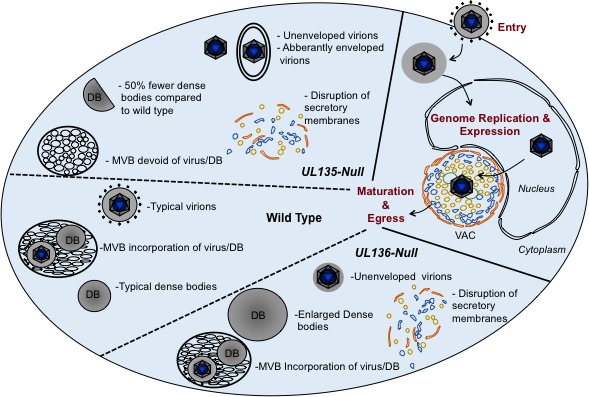
Viral replication in ECs and summary of phenotypes associated with UL135 and UL136 Viral determinants have been identified for entry and delivery of the genome to the nucleus and now for later stages of infection in ECs. During infection, secretory membranes (Golgi, endosomes, lysosomes, MVBs) are organized into a viral assembly compartment (VAC). CMV infection produces virions and dense bodies (DBs), which are incorporated into MVBs. *UL135*- and *UL136*-mutant virus infection results in the disruption of intracellular membrane organization and altered maturation of progeny virus.

The *UL133/8* locus contains four genes: *UL133*, *UL135*, *UL136*, and *UL138*. While *UL133* and *UL138* suppress viral replication for latency in CD34^+^ HPCs, *UL135* and *UL136* are required for replication in both CD34+ HPCs and ECs. The disruptions to intracellular membrane organization associated with the *UL133/8*-mutant virus infection in ECs largely segregate between *UL135*- and *UL136*-mutant viruses (Figure 1) [5, 7]. Viruses lacking *UL135* fail to incorporate virions in to MVBs, as occurs prominently in ECs infected with CMV. Viruses lacking *UL135* or *UL136* produce capsids that are aberrantly enveloped or lack their final envelope all together. Dense bodies, vesicles of viral tegument protein, are reduced in number or enlarged in size in *UL135*- and *UL136*-mutant virus infection, respectively. Further, infection with either *UL135*- or *UL136*-mutant virus reduces the reorganization of intracellular secretory membranes into the viral-induced assembly compartment—a hallmark of CMV infection. *UL136* encodes 5 protein isoforms, which have unique and differential effects on MVB biogenesis, assembly compartment formation and virus maturation in ECs (Caviness and Goodrum, unpublished results). Our work indicates that *UL135* and *UL136* function in co-opting cellular trafficking pathways for viral replication, but the mechanisms of their action and cooperation are not yet known. While the failure to reorganize or maintain organization of secretory membranes may ultimately be realized in defects in virion maturation and egress, the primary purpose of viral-mediated membrane organization and the alteration of MVBs may be to regulate trafficking, secretion or signaling.

While CMV encodes determinants specifically to ensure entry and delivery of the genome to the nucleus in ECs [8], UL135 and UL136 are the first viral determinants identified as important for post-entry tropism in ECs. Our studies illuminate complex interactions between viral proteins and the host trafficking machinery. We are just beginning to understand how a multitude of viruses commandeer host trafficking pathways for their replication and how this impacts cellular functions including signaling and secretion. Further investigation in this area will uncover strategies by which viruses ensure tropism in ECs and novel targets for antivirals to restrict replication in ECs, a reservoir critical to virus spread and pathogenesis.
